# “The Truth Is, We Must Miss Some”: A Qualitative Study of the Patient Eligibility Screening Process, and Automation Perspectives, for Cancer Clinical Trials

**DOI:** 10.1002/cam4.70466

**Published:** 2024-12-03

**Authors:** A. La Rosa, M. Vaterkowski, M. Cuggia, B. Campillo‐Gimenez, C. Tournigand, B. Baujat, C. Daniel, E. Kempf, G. Lamé

**Affiliations:** ^1^ Laboratoire d'Informatique Médicale et d'Ingénierie des Connaissances Pour la e‐Santé, LIMICS Sorbonne University, Inserm, Université Sorbonne Paris Nord Paris Cedex France; ^2^ LTSI‐UMR 1099 Université de Rennes, CHU de Rennes Rennes France; ^3^ LTSI‐UMR 1099 Université de Rennes, CLCC Eugène Marquis Rennes France; ^4^ Department of Medical Oncology, Henri Mondor and Albert Chenevier Teaching Hospital Université Paris Est Créteil, Assistance Publique—Hôpitaux de Paris Creteil France; ^5^ Department of Otorhinolaryngology‐Head and Neck Surgery Sorbonne University, Assistance Publique—Hôpitaux de Paris, Tenon Hospital Paris Cedex France; ^6^ Laboratoire de Génie Industriel, CentraleSupélec—Paris‐Saclay Campus Gif Sur Yvette France

**Keywords:** clinical decision support system, clinical trial, electronic health records, patient selection, qualitative research

## Abstract

**Background:**

Recruitment of cancer patients into clinical trials (CTs) is a challenge. We aimed to explore how patient eligibility assessment is conducted in practice, what factors support or hinder this process, and to assess the potential usefulness of Clinical Trial Recruitment Support Systems (CTRSS) for patient‐to‐trial matching.

**Methods:**

We conducted semi‐structured interviews in France with healthcare professionals involved in cancer CTs and experts on trial recruitment. We focused on the stages in‐between trial feasibility, and patient information and consent. Interviews were recorded, and the transcripts were analyzed thematically. We used the Systems Engineering Initiative for Patient Safety (SEIPS) 2.0 framework to organize our results.

**Results:**

We interviewed 25 participants. We identified common steps for cancer patient eligibility assessment: prescreening under medical supervision, followed by the validation of patient‐trial matching based on manual chart review. This process built on rich interactions between clinicians, other professionals (clinical research assistants, data scientists, medical coding experts), and patients. Technological factors, mainly related to data infrastructure (both for patient data and trial data), and organizational factors (research culture, incentives, formal and informal research networks) mediated the performance of the recruitment process. Participants had mixed feelings towards CTRSSs; they welcomed automated pre‐screening but insisted on manual verification. Given the necessary collaborative nature of multisite trials, coordinated efforts to support a common data infrastructure could be helpful.

**Conclusions:**

Material, organizational, and human factors affect cancer patient eligibility assessment for CTs. Patient‐to‐trial matching tools bear potential, but good understanding of the ecosystem, including stakeholders' motivations, is a prerequisite.

## 1 Introduction

Many clinical trials (CTs) in medical oncology struggle to recruit enough patients. Less than 10% of cancer patients are included in a CT [1], and 18% of CTs launched between 2000 and 2011 at the National Cancer Institute (NCI) closed with less than 50% of the target number of patients included after three or more years [2]. More concerning, another NCI study showed that around 20% of Phases II–IV cancer CTs failed to complete seven years after their launch, despite recruiting 48,000 patients [3]. This is a waste of research effort and hinders drug development and evaluation.

Patients are hardly responsible for this situation, since an estimated 85% of cancer patients in the United States are either unaware or unsure of the possibility to participate in CTs. When asked if they would have considered enrolling, 75% responded they would, had they known that it was possible [4].

The problem must then lie at the point of identifying patients that are eligible for a CT, a step commonly called “eligibility screening” [5]. This initial screening process accounts for 30%–40% of the time taken by the recruitment process [6, 7], and is broadly identified as a major reason for recruitment failure [8]. Although many studies have explored barriers related to patient information or CT design, which can complicate recruitment [9, 10, 11, 12, 13], the identification of eligible patients comes with its own challenges.

To streamline patient eligibility screening for CTs, researchers and industrials have started developing Clinical Trial Recruitment Support Systems (CTRSS) to automatically match patients with CTs, most often using Electronic Health Records (EHRs) [14, 15, 16, 17, 18, 19, 20, 21, 22, 23, 24, 25, 26]. Nonetheless, before deploying these solutions, it is crucial to understand the context, process, and work systems in which they would be integrated [27]. Since Fletcher and colleagues highlighted the potential of qualitative research for this purpose [28], qualitative studies of the patient recruitment process have flourished [8, 9, 11, 14, 15, 16, 17, 18, 19, 20, 21, 23, 24, 25, 26, 29]. In particular, qualitative studies have shown how patient screening still relied on significant manual effort, with minimal technological support [30, 31].

The objectives of this study were to characterize current screening processes and the factors that affect their performance, and to identify perspectives for facilitating cancer patient eligibility assessment for CTs, including through automatization. The study focuses on screening stages. We leave aside the upstream stage of feasibility assessment (i.e., before a trial is opened in a site) and the downstream stages of patient information, consent, and inclusion.

## 2 Material and Methods

We conducted a qualitative study in France, aiming to develop a qualitative description of patient eligibility screening [32]. We collected data through semi‐structured interviews in 2023. Participants were eligible for an interview if they were involved in cancer clinical research for over 5 years and involved in the process of cancer patient eligibility assessment for CTs. We identified participants through professional networks. We sampled purposefully to cover various roles in the CT screening process and various types of institutions. Participants were contacted by email and were sent the interview guide, consent form, and background information before the interview.

The interview guide was drawn up by a junior oncologist (A.L.R.), a senior medical oncologist and clinical researcher (E.K.), a biomedical informatician and physician (C.D.), and a health services researcher (G.L.) (Data S1). Participants were asked about their background, their current position, and their role in patient eligibility screening for cancer CTs. Their views on the current patient eligibility screening process and its local modalities, along with the positive and negative factors that influenced this process, were explored. Future perspectives, such as the implementation of CTRSS and the use of EHR data, were addressed. All participants provided written informed consent before interviews. Interviews were conducted in French by the first author (A.L.R.), a French junior oncologist without experience of recruitment in CTs and no prior relationship with interviewees, and lasted between 30 and 90 min. Interviews were audio‐recorded and transcribed verbatim.

We used conventional inductive content analysis to code the interview transcripts [33]. The analysis was informed by concepts drawn from the literature on recruitment for CTs, but also by the literature on human factors/ergonomics in healthcare. In particular, we used the Systems Engineering Initiative for Patient Safety (SEIPS) 2.0 framework to organize our findings [34]. The SEIPS framework is a tool to analyze the interactions between people, technology, tasks, organization, and environment in a work system. Those interactions enact processes, from which outcomes (desirable or not, short‐ or long‐term) emerge. The junior oncologist who conducted the interviews coded the meaning units and then grouped codes into sub‐themes and themes. We organized regular study team meetings to discuss the content of the interviews and the coding scheme. We used ATLAS.ti Mac (Version 23.1.1 (build 4239), ATLAS.ti, Berlin, Germany) to support the coding process.

This study was approved by the Paris‐Saclay Research Ethics Committee (CER‐Paris‐Saclay‐2023‐033). We used the SRQR guidelines to guide the reporting of the study [35].

## 3 Results

We contacted 31 people. Six did not respond to our e‐mails, and twenty‐five provided informed consent to participate. The participants were from various profiles and institutions (Table 1).

**TABLE 1:** Participant characteristics.

Participants characteristics	*N* = 25
Profile*	
Medical oncologist and PI	10
Clinical research assistant	5
Data scientist	2
Medical informatics physician	1
Statistician	1
Health sociologist	1
Representative of pharmaceutical industry	2
CTRSS or registry developer	4
Institution type*	
Not‐for‐profit comprehensive cancer center	10
Public university hospital	6
Not‐for‐profit private general hospital	3
Public general hospital	1
Pharmaceutical company	2
Software company	3
Drug regulation agency	3
National cancer institute	1
Geography	
Paris region	15
Outside of Paris region	10
Years of experience in clinical research	
Over 10 years	22
5–10 years	3
Gender	
Male	18
Female	7

Abbreviations: CTRSS, Clinical Trial Recruitment Support Systems; PI, principal investigator.

* Some participants matched multiple profiles, and some worked in more than one institution.

**FIGURE 1:**
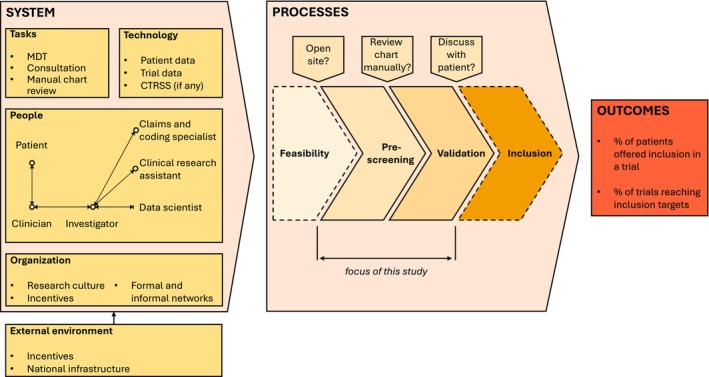
Work system for patient eligibility screening for clinical trials in oncology, mapped onto the SEIPS 2.0 framework [34]. Our study focuses on the ‘pre‐screening’ and ‘validation’ stages. We did not identify any element of the ‘internal environment’ affecting CT recruitment. CTRSS, Clinical trial recruitment support system; MDT, Multidisciplinary team meeting.

In the interviews, participants described the processes and the work system they currently experienced to reach the dual objective of offering participation in a trial to as many patients as possible and recruiting enough participants for all trials. We mapped these findings on the SEIPS 2.0 framework (Figure 1). In this section, we first discuss the factors by category (processes, tasks and people, technology, and organization, with ‘external environment’ factors distributed in the relevant other categories). We then turn to participants' views on automating eligibility screening processes.

### 3.1 Processes

We identified a common framework for cancer patient eligibility assessment. It has two main stages: prescreening and validation.

#### 3.1.1 Prescreening

The first step, called ‘prescreening’ by our interviewees, consists in a preliminary overall assessment of the patient eligibility based on a small set of criteria:

- Patient functional status
- Patient main comorbidities such as heart or kidney failure
- Patient biological data
- Site and staging of primitive tumor
- Localization of potential metastatic sites
- Whether the cancer is accessible to biopsy and measurable
- Previous anticancer treatment
- Tumor molecular profile if known

In one tertiary cancer comprehensive centers, this prescreening stage was formalized: a document containing the minimum dataset to assess patient eligibility is sent by the referring center and a dedicated meeting with all principal investigators takes place twice a week to prescreen all patients. In other places, when the treating oncologist is also the investigator of the trial, they are responsible for the prescreening step. When patients must be referred to another center for the trial, the prescreening step is managed conjointly between the clinician at the referring site and the trial investigator.

Whereas most of the data required for prescreening are available from the EHR or the anticancer treatment prescription tools, some criteria are prone to interobserver variability, for example, patient functional status. This could induce ‘compassionate’ inclusions, where a clinician thinks the patient will not pass the next screening stage, but still sends their data downstream. One participant argued that this criterion could be replaced by objective measures to avoid screening failure.

Because it's complicated to just tell patients ‘I have no objective criteria but I don't feel it’. There are times, we know it won't pass, but we still put them in. And we just wait and see. (Oncologist)

#### 3.1.2 Validation Through Manual Chart Review

The prescreening step is always followed by a thorough assessment of all the patient's record against all the CT inclusion criteria. Indeed, ‘in general, we get 60 inclusion criteria, exclusion criteria per trial, you won't find all 60 in the MDT report’ (Oncologist). Therefore, the full patient record must be reviewed, usually by a clinical research assistant (CRA) assisted by the investigator. Participants described this step as time‐consuming, and dependent on the quality of the prescreening stage. Then, if the patient is deemed eligible, they can be offered inclusion in the trial.

#### 3.1.3 Specific Cases

A number of specific cases to this process appeared in our interviews. The process above best represents Phase III/IV trials for second and following lines of treatment.

##### 3.1.3.1 Phases I/II, First Lines and Rare Cancers

Phases I and II CTs differ from Phase III/IV CTs. First, because few slots are open, over a very short period. This means that the list of Phase I/II trials open in a center is difficult to update, since it changes frequently. Second, the time constraint creates competition between participating centers to open the trial as quickly as possible. While phase III trials sometimes struggle to include patients, phase I/II trials do not usually face such an issue. Clinicians reported the opposite concern: going fast to offer as many of their patients as possible to enter the trial.

For phase I trials, honestly, it changes so much, we're back to this thing where people tell me ‘could you update the trial list’, but honestly … We've given up on doing this. We meet every week, and every week, it changes. (Oncologist)

For CTs of first line treatments, centers must usually rely on their own internal recruitment. Indeed, patients referred from elsewhere are likely to have already undergone some form of treatment. This is why first line trials tend to be open in large tertiary centers.

‘Rare’ cancers are also specific, because CTs more often fulfill an unmet medical need for those patients. Therefore, even with a low prevalence, a center that opens a trial for a rare cancer usually meets its objectives, because there are few alternatives and high demand. CTs for rare cancers also benefit from specific research networks focused on rare cancers.

##### 3.1.3.2 Biomolecular‐Based CT Recruitment

With the emergence of biomolecular tumor data and targeted treatment, more and more trials integrate molecular criteria in their design, which leads to very restricted populations.

It's much more fragmented than before, we used to have studies, you could get 50 patients in one place. Now, it's much more segmented in oncology, with molecular biology, with all the available technologies. (Oncologist)

Biomolecular screening of the tumor is not always incorporated in routine care. Hence, most patients need an extra biopsy or a re‐analysis of a previous biopsy to check for the targeted molecular alteration. This means more time and steps for patients, and sometimes the need to screen a huge number of patients to find the target biomolecular anomaly. Thus, some participants advocated generalized, routine upstream molecular testing to facilitate patient identification. Having a team dedicated to this process was seen as a promising perspective.

### 3.2 Roles, Tasks, and Interpersonal Interactions

Figure 1 illustrates the interactions between six roles in the patient eligibility screening process: the patient, the clinician, the investigator, the CRA, the data scientist, and the medical coding expert. We now detail the content of these interactions.

#### 3.2.1 On the Frontline: Clinicians and Patients

The only actors involved in both routine care and in research (potentially) are the clinician and the patient. The clinician holds a central role in CTs, as they are the sole bridge between routine care and research. Depending on the institution in which they work, they can also be the investigator for some trials.

The clinician's role in the recruitment process is to identify which of their patients could be eligible for the trials currently open. Interviewees mentioned that always keeping in mind the list of open CTs, while managing patients, generated an intense cognitive load, leading to mishaps.

The truth is, we must miss some. Our memory is not extensible. (Oncologist)

Inclusion in a trial can be discussed during the consultation, or the clinician can make a note that they need to investigate this offline after the consultation. Clinicians can also discuss CT inclusion during MDTs, where treatment options are assessed collectively. Regional, pathology‐specific, or molecular MDTs were identified as key opportunities for recruitment. Referrals for a second opinion also sometimes led to proposals of trial inclusion.

All along the care and recruitment process, clinicians need to be mindful of time. Cancer patients are classically screened for CT eligibility when they reach the end of therapeutic options. However, the question of inclusion in a trial sometimes arose earlier: from the first line of treatment in cancers with poor prognosis, or at every stage of the disease for some oncologists who were more inclined to consider trials as a treatment option−for example, those involved in clinical research. Time was all the more important for rapidly progressing diseases. Clinicians highlighted that it was important to discuss trial inclusion early with the patient, because it might be difficult to sensitize the patient to clinical research later on, or because they might then already be engaged in another protocol. In fact, patients seem to decide on their participation before getting to the information and consent stage.

The decision work for the patient, to participate or not in a trial … Our hypothesis, it's that it's not at the moment of signing consent, it generally happens before, with the referring clinician, without them having information on the trial in which they could be included. (Sociologist)

In the trial timeline, the identification of eligible patients is performed once during the feasibility study (when investigators consider sites for inclusion). Then, it should continue as long as the trial remains open. However, some participants mentioned that as time passed, clinicians tended to include less because they forgot that the trial was open for inclusion.

Overall, the screening process was described by participants as “hand‐made”, “artisanal” and relying heavily on the clinician's constant attention to open CTs and awareness of the need to consider inclusion for their patients. Some interviewees indicated ways to support this process. For example, local investigators can reverse the process and look for open trials for their current patients. If they have a good ‘potential’ in a certain category of patients, they can then proactively try to open a trial at their hospital for these patients.

For example, you realize that in 2021–2022, you've had many cases of prostate cancer, bladder cancer and you haven't offered them a possible trial. Then, you know that you have potential or at least you have a need, you can turn the question on its head, it's not that you have potential for a trial, it's that you have a need from patients to get into trials. Then the job is to go out to find the trial that fits the needs you have. (Oncologist)

Patients are often seen as passive in the recruitment process, but interviewees described patients being more and more informed about clinical research. Some patients spontaneously mentioned CTs as a treatment option to their doctor or, even, for a small number of well‐informed patients, actively searched available CTs by directly contacting CT investigators. Participants generally viewed patient autonomation positively.

#### 3.2.2 Orchestrating Trial Recruitment: The Investigator

The CT investigator is ultimately responsible for the whole screening process. Their involvement is crucial as it conditions the success of a trial. This success depends on (1) the investigator's evaluation of recruitment potential when opening recruitment sites, along with awareness of competing trials, and (2) sustained motivation during the inclusion step to maintain satisfying patient recruitment during the whole duration of the trial.

The list of patients is one element, but then, the big thing is the involvement of the coordinating investigator and of the main investigators in the centers, who work hard or don't … Then you need public research resources, meaning CRAs, to help with recruitment, of course they're as important. Each pillar is crucial. If just one link breaks, is not good, it can… It's the weak link in fact and it means that after that, there's no recruitments. (Statistician)

Some participants estimated that up to 70% of patients included in CTs came from other centers. Investigators and clinicians reported that the main method to ensure external referral is through interpersonal connections. Interviewees explained that existing formal or informal networks facilitated recruitment for members of the network, but could also hinder it for researchers outside of the network.

These are quite informal networks based on personal relationships, obviously … you manage the best you can, but there is no formal network that guarantees that patients get referred to trials. (Oncologist)

Aside from personal networks, built through training, common projects, and professional experience, some tertiary centers benefit from a national reputation that allows them to recruit patients from all over the territory. Geographical research and referral networks also exist in France. Investigators described them as heterogeneous in terms of spatial repartition and organization. Yet, promotors favored sites that are embedded in a geographical network to facilitate referrals and avoid relying solely on interpersonal connections. Pathology networks were pictured as well organized and effective for rare cancers but less helpful for more prevalent diseases.

#### 3.2.3 The Back Office: Clinical Research Assistants

Participants described the interaction between investigators and CRAs in depth during the interviews. Investigators usually contact the CRA when a patient could be eligible, so that the CRA can review the patient's EHR in detail. The CRA is also responsible for reminding investigators of which trials are open to recruitment in which indications. They can facilitate awareness of CT characteristics by participating in MDTs, meeting with clinicians to discuss trials, providing CT summary booklets in consultation, or participating in clinical research meetings to inform the medical team of available trials. CRAs can participate in the prescreening step alongside clinicians, for example, by reviewing all patient files before they are discussed in MDTs, or before consultations. These procedures can be routine or used as a last resort if a trial is struggling to include enough patients.

The level of involvement varies depending on the CRA, and on how this role integrates in their job arrangements. In some structures, CRAs are nurses and intervene both in care and in research.

I have a look at MDTs, I check them afterwards, I re‐screen them and because there are often patients that get overlooked and I look at the criteria, etc. And I talk to the clinician before the consultation. To say ‘I think they're eligible even if they haven't been identified in the MDT.’… I find a lot. Depending on the study, more than half. Because indeed, for some studies they think about it, for others they rely a bit on me too. (CRA)

An oncologist even proposed that regional or national platforms that organize joint MDTs (e.g., molecular MDTs or regional MDTs) could have their own CRA to help with patient referral.

### 3.3 Technological Factors

Screening patients requires assessing if a patient fills a CT's eligibility criteria. This task requires good quality data on both sides (patient and trial). In practice, such data is often not available or not readily usable in the right format.

#### 3.3.1 Patient Data

Patient data is accessible mainly through the EHR, through anticancer treatment prescription systems, and sometimes through local patient registries. EHR data is very exhaustive but it is mainly unstructured, except for demographic data, and diagnosis and procedure codes, which are not immediately available. Local patient registries are well structured and curated, but updated with variable delays. Latency in updating EHRs and registries is of particular concern for patients with progressive diseases.

Anticancer treatment prescription tools only provide key features, such as age, weight, tumor type, stage, and prescribed medication. Some investigators reported that they often used prescription tools for feasibility studies or to prescreen patients if a CT did not fulfill its inclusion goal.

Data scientists and claims coding experts can be called upon by the investigator to help pre‐identify target patients, or to assess recruitment potential. They possess data expertise that can be beneficial to this task. However, their involvement is not systematic and depends on how available these people are for clinical research.

#### 3.3.2 Trial Data

CT characteristics are accessible through local, national, or international databases. Oncologists said that they rarely use national or international databases because trial data are poorly structured, and updates are not frequent enough. Local databases are not maintained in all institutions.

Therefore, the characteristics of ongoing or upcoming trials are often discussed through informal channels, in internal clinical research meetings and during congresses or regional MDTs. The quality and standardization of CT inclusion criteria data was a challenge for interviewees.

### 3.4 Organizational Factors

Organizational factors affected CT recruitment processes. First, because clinicians differed in their vision of trials, with some seeing it as an integral part of the patient pathway, while others saw it as separate from regular care.

There are still people, and I think it's a pity, who consider that clinical research is not necessarily something that's part of the patient pathway. But I think it's changing. (Oncologist)

Second, participants highlighted how opinions varied on what counted as a satisfactory rate for patient inclusion in trials.

We can't get a fivefold increase, but if we doubled the number of patients we include, it would be huge. My own projection is that clinicians are in a form of self‐satisfaction on how many patients we include whereas we could go much further so there's room for improvement. (Oncologist)

CRAs, data scientists, and epidemiologists were prone to point out the flaws of ‘hand‐made processes’. Oncologists showed more satisfaction. Participants working in comprehensive cancer centers reported more satisfaction with current recruitment processes than those working in university hospitals or general hospitals.

Participants described a health system that does not incentivize the work required to manage trial recruitment. Clinicians get no incentive to discuss trials with patients. Since hospitals are funded based on their activity, referring a patient to another center means a net loss for the referring hospital. Some participants also described recruitment networks segmented by clinical specialty (e.g., medical oncology, surgery, radiotherapy), or by organization status (public vs. private). Finally, resources and routines are best available in departments that do more CTs.

To challenge the status‐quo, one interviewee suggested that inclusion in trials should be a key quality indicator in oncology departments, just as the percentage of patients discussed in MDTs is monitored.

### 3.5 Perspectives on Automation

#### 3.5.1 Towards Automated Prescreening?

Most participants saw the perspective of automating part of the recruitment process with enthusiasm, although oncologists who are already satisfied with the current process showed less enthusiasm. All agreed on focusing automation of the prescreening step while keeping the final review manual.

Two approaches emerged from interviews. First, a ‘patient‐centered’ approach (i.e., find trials for a patient), the most frequently discussed by interviewees. This could consist in an eligibility alert during consultation or MDT to inform the physician that their patient fits prescreening criteria for one or several trials. Clinicians described pop‐up windows integrated in the EHR, the anticancer treatment prescription tool, or the MDT management tool. Most participants would prefer this integrated solution over a distinct software where they would need to manually enter patient data−the approach chosen by the three developers that we interviewed.

The other approach is ‘trial‐centered’ (i.e., find patients for a trial). It consists in checking which patients could be included for a given CT. Some centers already do this when trials fail to include, or during feasibility studies. Some participants saw the main benefit of a CTRSS in feasibility studies.

Participants mentioned that any attempt at deploying a CTRSS would face issues of data interoperability between centers. Besides, a CTRSS would need to build on a regularly updated list of CTs, something clinicians said they could also use outside of a CTRSS.

What would make life easier for me, would really be a tool where you know in real time, even in a consultation, now we're used to going on Google and all that. So, just something that lists everything that's open and not open. (Oncologist)

In any approach, participants highlighted that the accuracy of a tool would be key to success. Alarm fatigue was already anticipated as a challenge.

If there's too many false positives, after a while, people give up, I suppose. When the things pop up and ‘but this patient it's not it does not fit at all I'll never include them’. After a while people don't even look any more, that's the problem. (Statistician)

#### 3.5.2 CTRSS Users and Patient Involvement

Most participants thought any CTRSS should be designed for medical use as the physician is the main actor in prescreening. However, some suggested that CRAs could also use the tool, especially before consultations or MDTs. Participants highlighted the need for IT supervision and data expertise, but noted that the lack of human and financial resources for CT management could hamper the deployment of new solutions.

Interviewees shared a wish to better inform patients about CTs, but had mixed views on patient access to CTRSS. On the one hand, patients and patient organizations increasingly ask to be involved in CT recruitment, and all CTRSS developers we talked to targeted patients as potential users. On the other hand, interviewees reported that whereas some patients are well informed about their disease and therapeutic options, the majority do not have this kind of expertise and rely heavily on their physician. Free access to pre‐screening tools without medical support could generate false hopes and frustration for patients. Finally, uncoordinated efforts by patients and doctors looking simultaneously for a CT could be a waste of energy on both sides.

## 4 Discussion

### 4.1 Findings

From interviews with 25 French participants involved in recruitment for CTs in oncology, we used the SEIPS 2.0 framework to describe the current recruitment system and processes.

Interviewees described a two‐stage manual process to pre‐screen and validate patient records for inclusion. The recruitment system relies heavily on personal networks and on clinicians' constant attention to ongoing trials during clinical activities, especially consultations and MDTs. CRAs are central in this effort to identify as many eligible patients as possible, but their presence and availability vary, leading to inconsistencies in recruitment effectiveness across sites. Data is central to prescreening and validation efforts, but is handled in an ad‐hoc fashion, with little dedicated solutions. Processes and routines differed according to the level of resources of hospitals, from generalized molecular testing with multiple CRAs in some tertiary centers, to clinicians recruiting individually in smaller centers.

Participants showed enthusiasm for automating the prescreening stage but remained committed to a final manual review. For participants, these systems should take the clinician's perspective—faced with a patient, what trial could I discuss with them?—rather than the trial‐centered approach—given a trial, which of my patients could be included?

### 4.2 Strengths and Limitations

These results were obtained from a broad sample of participants, representing all types of public and non‐profit institutions involved in clinical research in France. We used standard qualitative research procedures, and a well‐established framework to organize findings (SEIPS 2.0). The study team discussed results collectively in regular meetings, but we did not ask interviewees to check transcripts or validate our interpretations. Notably, we did not include patients or patient representatives in our participants. Others have focused on patients, but at later stages, after screening. Our interviews suggest that at this stage, patients are not involved in upstream stages, and future research could focus on if and how they could be involved in these stages, for example, through participatory or open science methods [36, 37].

### 4.3 Interpretation

Our depiction of a mostly manual, two‐stage recruitment process matches previous reports [30, 31, 38, 39, 40]. The central role of, and the challenges encountered by, clinicians in recruiting patients have also been underlined before [41, 42, 43, 44]. Finally, the role of MDTs and collective engagement has been identified in previous qualitative research [29]. However, our joint investigation of current processes and automation perspectives brings new insights.

Even if the current ‘manual’ process works for some clinicians, it seems to have reached its limit in terms of inclusion rate and delay. Relying entirely on clinicians to identify suitable patients can create unconscious biases in recruitment [45], and creates additional work for already overburdened clinicians [46, 47]. Research and clinical teams are still often separate [48], and having research tasks rely on clinicians who do not get rewarded or incentivized for performing them is likely to lead to suboptimal results. This means that recruitment relies heavily on the motivation of clinicians to include, which may understandably vary, given the lack of incentive and the demands of care.

Automation through CTRSSs could help tackle unconscious biases and identify more patients. The development of Clinical Data Warehouses, regularly populated by EHR data and accessible in conformance with interoperability standards, could help [49], although it comes with its own challenges [50]. Research on automated analysis of eligibility criteria is also steadily developing [21, 51, 52], and various CTRSSs for cancer CT have been described in the literature [15, 16, 18, 19, 20, 23, 26, 53, 54]. However, there is still little real life evaluation of the performance of these systems and of their impact on recruitment rate, delay, and workload.

Our results show that professionals welcome these developments. While previous studies have identified similar challenges in recruitment processes, our work also shows a common shared wish for the development of patient‐centered, automated prescreening systems to support the screening process. This vision includes the integration of automated tools into EHR systems and support for clinician decision‐making via real‐time notifications at critical points of care.

Automation raises questions about the availability of structured data for prescreening, the integration of these tools into existing workflows, and the acceptability of automation within the clinical decision‐making process. Most data in EHRs remain unstructured. Emerging technologies, including conversational AI models, may play a role in overcoming data limitations by assisting in the interpretation and specification of eligibility criteria. These tools could act as intermediaries, improving data quality and ensuring that clinicians have the necessary information to make informed decisions. However, acceptability remains key: clinicians and CRAs should be able to trust these systems to reduce false positives, with CRAs playing a vital role in reviewing and validating prescreening results. A CTRSS can only partly replace the central role of CRAs in coordinating efforts and engaging with frontline clinicians. More research needs to be done to understand who CTRSSs should target, and for what specific task.

All hospitals today are not even when it comes to research resources [55]. However, as inclusion criteria become more specific, even highly specialized tertiary centers will need to build on ever larger populations to identify eligible patients for their trials. Aside from technical fixes, such as CTRSSs, there is probably a role for higher‐level initiatives (regional, national) to structure the recruitment system, through incentives, joint structures with dedicated resources (CRAs, data infrastructure), and an up‐to‐date information system dedicated to trials.

## 5 Conclusion

Identifying patients who are eligible for trials is more complex than it seems and relies on a network of people supported by data and information technology. A common process exists, with variations between sites. Automation holds promises, but might best work on the pre‐screening stage, while keeping an ultimate human verification of patient records. Qualitative human factors research can help better understand this system.

## Author Contributions

Conceptualization: A. La Rosa, M. Vaterkowski, M. Cuggia, B. Campillo‐Gimenez, C. Daniel, E. Kempf, G. Lamé. Methodology: A. La Rosa, G. Lamé. Data curation: A. La Rosa. Investigation: A. La Rosa, M. Vaterkowski, M. Cuggia, B. Campillo‐Gimenez, C. Tournigand, B. Baujat, C. Daniel, E. Kempf. Validation: A. La Rosa, M. Vaterkowski, M. Cuggia, B. Campillo‐Gimenez, C. Tournigand, B. Baujat, C. Daniel, E. Kempf, G. Lamé. Formal analysis: A. La Rosa. Supervision: C. Tournigand, B. Baujat, C. Daniel, E. Kempf, G. Lamé, Funding acquisition: B. Baujat. Visualization: A. La Rosa, G. Lamé, Project administration: A. La Rosa, C. Daniel, E. Kempf. Resources: A. La Rosa, G. Lamé. Writing – original draft: A. La Rosa. Writing – review and editing: A. La Rosa, M. Vaterkowski, M. Cuggia, B. Campillo‐Gimenez, C. Tournigand, B. Baujat, C. Daniel, E. Kempf, G. Lamé.

## Conflicts of Interest

The authors declare no conflicts of interest.

## Supporting information


Data S1.

## Data Availability

The data that support the findings of this study are available on request from the corresponding author. The data are not publicly available due to privacy or ethical restrictions.

## References

[1] M. N. Fouad , J. Y. Lee , P. J. Catalano , et al., “Enrollment of Patients With Lung and Colorectal Cancers Onto Clinical Trials,” Journal of Oncology Practice 9, no. 2 (2013): e40–e47. 23814523 10.1200/JOP.2012.000598 PMC3595449

[2] C. S. Bennette , S. D. Ramsey , C. L. McDermott , J. J. Carlson , A. Basu , and D. L. Veenstra , “Predicting Low Accrual in the National Cancer Institute's Cooperative Group Clinical Trials,” Journal of the National Cancer Institute 108, no. 2 (2016): djv324. 26714555 10.1093/jnci/djv324 PMC5964887

[3] K. D. Stensland , R. B. McBride , A. Latif , et al., “Adult Cancer Clinical Trials That Fail to Complete: an Epidemic?,” Journal of the National Cancer Institute 106, no. 9 (2014): dju229. 25190726 10.1093/jnci/dju229

[4] R. L. Comis , J. D. Miller , C. R. Aldigé , L. Krebs , and E. Stoval , “Public Attitudes Toward Participation in Cancer Clinical Trials,” Journal of Clinical Oncology 21, no. 5 (2003): 830–835. 12610181 10.1200/JCO.2003.02.105

[5] L. T. Penberthy , B. A. Dahman , V. I. Petkov , and J. P. DeShazo , “Effort Required in Eligibility Screening for Clinical Trials,” Journal of Oncology Practice 8, no. 6 (2012): 365–370. 23598846 10.1200/JOP.2012.000646 PMC3500483

[6] J. W. Dexheimer , H. Tang , A. Kachelmeyer , et al., “A Time‐and‐Motion Study of Clinical Trial Eligibility Screening in a Pediatric Emergency Department,” Pediatric Emergency Care 35, no. 12 (2019): 868–873. 30281551 10.1097/PEC.0000000000001592 PMC6445787

[7] Y. Ni , M. Bermudez , S. Kennebeck , S. Liddy‐Hicks , and J. Dexheimer , “A Real‐Time Automated Patient Screening System for Clinical Trials Eligibility in an Emergency Department: Design and Evaluation,” JMIR Medical Informatics 7, no. 3 (2019): e14185. 31342909 10.2196/14185 PMC6685132

[8] M. Briel , K. K. Olu , E. von Elm , et al., “A Systematic Review of Discontinued Trials Suggested That Most Reasons for Recruitment Failure Were Preventable,” Journal of Clinical Epidemiology 80 (2016): 8–15. 27498376 10.1016/j.jclinepi.2016.07.016

[9] A. Bill‐Axelson , A. Christensson , M. Carlsson , B. J. Norlén , and L. Holmberg , “Experiences of Randomization: Interviews With Patients and Clinicians in the SPCG‐IV Trial,” Scandinavian Journal of Urology and Nephrology 42, no. 4 (2008): 358–363. 19230168 10.1080/00365590801950253

[10] D. B. Fogel , “Factors Associated With Clinical Trials That Fail and Opportunities for Improving the Likelihood of Success: A Review,” Contemporary Clinical Trials Communications 11 (2018): 156–164. 30112460 10.1016/j.conctc.2018.08.001 PMC6092479

[11] D. W. Hamilton , I. de Salis , J. L. Donovan , and M. Birchall , “The Recruitment of Patients to Trials in Head and Neck Cancer: A Qualitative Study of the EaStER Trial of Treatments for Early Laryngeal Cancer,” European Archives of Oto‐Rhino‐Laryngology 270, no. 8 (2013): 2333–2337. 23334205 10.1007/s00405-013-2349-8

[12] E. J. Mills , D. Seely , B. Rachlis , et al., “Barriers to Participation in Clinical Trials of Cancer: A Meta‐Analysis and Systematic Review of Patient‐Reported Factors,” Lancet Oncology 7, no. 2 (2006): 141–148. 16455478 10.1016/S1470-2045(06)70576-9

[13] N. Mills , J. M. Blazeby , F. C. Hamdy , et al., “Training Recruiters to Randomized Trials to Facilitate Recruitment and Informed Consent by Exploring patients' Treatment Preferences,” Trials 15, no. 1 (2014): 323. 25115160 10.1186/1745-6215-15-323 PMC4138384

[14] M. Ansoborlo , T. Dhalluin , C. Gaborit , M. Cuggia , and L. Grammatico‐Guilllon , “Prescreening in Oncology Using Data Sciences: The PreScIOUS Study,” Studies in Health Technology and Informatics 281 (2021): 123–127. 34042718 10.3233/SHTI210133

[15] J. T. Beck , M. Rammage , G. P. Jackson , et al., “Artificial Intelligence Tool for Optimizing Eligibility Screening for Clinical Trials in a Large Community Cancer Center,” JCO Clinical Cancer Informatics 4 (2020): 50–59. 31977254 10.1200/CCI.19.00079

[16] D. Calaprice‐Whitty , K. Galil , W. Salloum , A. Zariv , and B. Jimenez , “Improving Clinical Trial Participant Prescreening With Artificial Intelligence (AI): A Comparison of the Results of AI‐Assisted vs Standard Methods in 3 Oncology Trials,” Therapeutic Innovation & Regulatory Science 54, no. 1 (2020): 69–74. 32008227 10.1007/s43441-019-00030-4

[17] O. Gangl , K. Sahora , P. Kornprat , et al., “Preparing for Prospective Clinical Trials: A National Initiative of an Excellence Registry for Consecutive Pancreatic Cancer Resections,” World Journal of Surgery 38, no. 2 (2014): 456–462. 24121365 10.1007/s00268-013-2283-3

[18] D. A. Hanauer , J. S. Barnholtz‐Sloan , M. F. Beno , et al., “Electronic Medical Record Search Engine (EMERSE): An Information Retrieval Tool for Supporting Cancer Research,” JCO Clinical Cancer Informatics 4 (2020): 454–463. 32412846 10.1200/CCI.19.00134 PMC7265780

[19] A. Hein , P. Gass , C. B. Walter , et al., “Computerized Patient Identification for the EMBRACA Clinical Trial Using Real‐Time Data From the PRAEGNANT Network for Metastatic Breast Cancer Patients,” Breast Cancer Research and Treatment 158, no. 1 (2016): 59–65. 27283834 10.1007/s10549-016-3850-8

[20] H. Huebner , C. M. Kurbacher , G. Kuesters , et al., “Heregulin (HRG) Assessment for Clinical Trial Eligibility Testing in a Molecular Registry (PRAEGNANT) in Germany,” BMC Cancer 20, no. 1 (2020): 1091. 33176725 10.1186/s12885-020-07546-1 PMC7656772

[21] B. Idnay , C. Dreisbach , C. Weng , and R. Schnall , “A Systematic Review on Natural Language Processing Systems for Eligibility Prescreening in Clinical Research,” Journal of the American Medical Informatics Association 29, no. 1 (2021): 197–206. 34725689 10.1093/jamia/ocab228 PMC8714283

[22] L. R. Kalankesh and E. Monaghesh , “Utilization of EHRs for Clinical Trials: A Systematic Review,” BMC Medical Research Methodology 24, no. 1 (2024): 70. 38494497 10.1186/s12874-024-02177-7 PMC10946197

[23] J. Kamal , K. Pasuparthi , P. Rogers , J. Buskirk , and H. Mekhjian , “Using an Information Warehouse to Screen Patients for Clinical Trials: A Prototype,” American Medical Informatics Association Annual Symposium Proceedings 2005 (2005): 1004. PMC1560829 16779291

[24] A. Kotoulas , G. Lambrou , and D.‐D. Koutsouris , “Design and Virtual Implementation of a Biomedical Registry Framework for the Enhancement of Clinical Trials: Colorectal Cancer Example,” BMJ Health & Care Informatics 26, no. 1 (2019): e100008. 10.1136/bmjhci-2019-100008 PMC7062330 31142494

[25] D. F. Patrão , M. Oleynik , F. Massicano , and S. A. Morassi , “Recruit—An Ontology Based Information Retrieval System for Clinical Trials Recruitment,” Studies in Health Technology and Informatics 216 (2015): 534–538. 26262108

[26] B. J. Rimel , J. Lester , L. Sabacan , et al., “A Novel Clinical Trial Recruitment Strategy for Women's Cancer,” Gynecologic Oncology 138, no. 2 (2015): 445–448. 26001329 10.1016/j.ygyno.2015.05.008

[27] P. V. Kukhareva , C. Weir , G. Del Fiol , et al., “Evaluation in Life Cycle of Information Technology (ELICIT) Framework: Supporting the Innovation Life Cycle From Business Case Assessment to Summative Evaluation,” Journal of Biomedical Informatics 127 (2022): 104014. 35167977 10.1016/j.jbi.2022.104014 PMC8959015

[28] B. Fletcher , A. Gheorghe , D. Moore , S. Wilson , and S. Damery , “Improving the Recruitment Activity of Clinicians in Randomised Controlled Trials: A Systematic Review,” BMJ Open 2, no. 1 (2012): e000496. 10.1136/bmjopen-2011-000496 PMC3253423 22228729

[29] S. Strong , S. Paramasivan , N. Mills , C. Wilson , J. L. Donovan , and J. M. Blazeby , “'The Trial Is Owned by the Team, Not by an Individual': A Qualitative Study Exploring the Role of Teamwork in Recruitment to Randomised Controlled Trials in Surgical Oncology,” Trials 17, no. 1 (2016): 212. 27113592 10.1186/s13063-016-1341-1 PMC4845366

[30] L. Becker , T. Ganslandt , H. U. Prokosch , and A. Newe , “Applied Practice and Possible Leverage Points for Information Technology Support for Patient Screening in Clinical Trials: Qualitative Study,” JMIR Medical Informatics 8, no. 6 (2020): e15749. 32442156 10.2196/15749 PMC7327588

[31] K. Fitzer , R. Haeuslschmid , R. Blasini , et al., “Patient Recruitment System for Clinical Trials: Mixed Methods Study About Requirements at Ten University Hospitals,” JMIR Medical Informatics 10, no. 4 (2022): e28696. 35442203 10.2196/28696 PMC9069280

[32] M. Sandelowski , “Whatever Happened to Qualitative Description?,” Research in Nursing & Health 23, no. 4 (2000): 334–340. 10940958 10.1002/1098-240x(200008)23:4<334::aid-nur9>3.0.co;2-g

[33] H.‐F. Hsieh and S. E. Shannon , “Three Approaches to Qualitative Content Analysis,” Qualitative Health Research 15, no. 9 (2005): 1277–1288. 16204405 10.1177/1049732305276687

[34] R. J. Holden , P. Carayon , A. P. Gurses , et al., “SEIPS 2.0: A Human Factors Framework for Studying and Improving the Work of Healthcare Professionals and Patients,” Ergonomics 56, no. 11 (2013): 1669–1686. 24088063 10.1080/00140139.2013.838643 PMC3835697

[35] B. C. O'Brien , I. B. Harris , T. J. Beckman , D. A. Reed , and D. A. Cook , “Standards for Reporting Qualitative Research: A Synthesis of Recommendations,” Academic Medicine 89, no. 9 (2014): 1245–1251. 24979285 10.1097/ACM.0000000000000388

[36] A. Hughes‐Morley , M. Hann , C. Fraser , et al., “The Impact of Advertising Patient and Public Involvement on Trial Recruitment: Embedded Cluster Randomised Recruitment Trial,” Trials 17, no. 1 (2016): 586. 27931252 10.1186/s13063-016-1718-1 PMC5146878

[37] A. Price , S. M. Liew , J. Kirkpatrick , J. Price , T. Lopreto , and Y. Nelken , “Mind the Gap in Clinical Trials: A Participatory Action Analysis With Citizen Collaborators,” Journal of Evaluation in Clinical Practice 23, no. 1 (2017): 178–184. 27917564 10.1111/jep.12678

[38] M. R. Cowie , J. I. Blomster , L. H. Curtis , et al., “Electronic Health Records to Facilitate Clinical Research,” Clinical Research in Cardiology 106, no. 1 (2017): 1–9. 10.1007/s00392-016-1025-6 PMC5226988 27557678

[39] R. M. Jimenez‐Rodriguez , G. Martín‐Gutiérrez , S. Jiménez‐Jorge , et al., “Factors Associated With Recruitment Success in the Phase 2a Study of Aztreonam–Avibactam Development Programme: A Descriptive Qualitative Analysis Among Sites in Spain,” BMJ Open 12, no. 2 (2022): e051187. 10.1136/bmjopen-2021-051187 PMC8814749 35115349

[40] C. Wilson , L. Rooshenas , S. Paramasivan , et al., “Development of a Framework to Improve the Process of Recruitment to Randomised Controlled Trials (RCTs): The SEAR (Screened, Eligible, Approached, Randomised) Framework,” Trials 19, no. 1 (2018): 50. 29351790 10.1186/s13063-017-2413-6 PMC5775609

[41] C. Clement , L. E. Selman , P. G. Kehoe , B. Howden , J. A. Lane , and J. Horwood , “Challenges to and Facilitators of Recruitment to an Alzheimer's Disease Clinical Trial: A Qualitative Interview Study,” Journal of Alzheimer's Disease 69 (2019): 1067–1075. 10.3233/JAD-190146 PMC6598018 31156168

[42] D. J. Einstein , “Compassion and Compassionate Use,” Journal of Clinical Oncology 36, no. 29 (2018): 2969–2971. 30156981 10.1200/JCO.18.00205 PMC7098837

[43] D. Elliott , S. Husbands , F. C. Hamdy , L. Holmberg , and J. L. Donovan , “Understanding and Improving Recruitment to Randomised Controlled Trials: Qualitative Research Approaches,” European Urology 72, no. 5 (2017): 789–798. 28578829 10.1016/j.eururo.2017.04.036

[44] R. J. Winn , “Obstacles to the Accrual of Patients to Clinical Trials in the Community Setting,” Seminars in Oncology 21, no. 4 Suppl 7 (1994): 112–117. 8091236

[45] C. Conefrey , J. L. Donovan , R. C. Stein , et al., “Strategies to Improve Recruitment to a De‐Escalation Trial: A Mixed‐Methods Study of the OPTIMA Prelim Trial in Early Breast Cancer,” Clinical Oncology 32, no. 6 (2020): 382–389. 32089356 10.1016/j.clon.2020.01.029 PMC7246331

[46] M. Dixon‐Woods , C. Summers , M. Morgan , and K. Patel , “The Future of the NHS Depends on Its Workforce,” BMJ 384 (2024): e079474. 38538029 10.1136/bmj-2024-079474

[47] J. Lawton , J. Kirkham , D. White , D. Rankin , C. Cooper , and S. Heller , “Uncovering the Emotional Aspects of Working on a Clinical Trial: A Qualitative Study of the Experiences and Views of Staff Involved in a Type 1 Diabetes Trial,” Trials 16, no. 1 (2015): 3. 25566971 10.1186/1745-6215-16-3 PMC4326295

[48] D. E. Gerber , T. Reimer , E. L. Williams , et al., “Resolving Rivalries and Realigning Goals: Challenges of Clinical and Research Multiteam Systems,” Journal of Oncology Practice 12, no. 11 (2016): 1020–1028. 27624948 10.1200/JOP.2016.013060 PMC5455413

[49] E. Kempf , M. Vaterkowski , D. Leprovost , et al., “How to Improve Cancer Patients ENrollment in Clinical Trials From rEal‐Life Databases Using the Observational Medical Outcomes Partnership Oncology Extension: Results of the PENELOPE Initiative in Urologic Cancers,” JCO Clinical Cancer Informatics 7 (2023): e2200179. 37167578 10.1200/CCI.22.00179

[50] S. Priou , E. Kempf , M. Jankovic , and G. Lamé , ““Goldmine” or “Big Mess”? An Interview Study on the Challenges of Designing, Operating, and Ensuring the Durability of Clinical Data Warehouses in France and Belgium,” Journal of the American Medical Informatics Association 31, no. 11 (2024): 2699–2707. 39269930 10.1093/jamia/ocae244 PMC11491596

[51] Q. Su , G. Cheng , and J. Huang , “A Review of Research on Eligibility Criteria for Clinical Trials,” Clinical and Experimental Medicine 23, no. 6 (2023): 1867–1879. 36602707 10.1007/s10238-022-00975-1 PMC9815064

[52] C. Weng , S. W. Tu , I. Sim , and R. Richesson , “Formal Representation of Eligibility Criteria: A Literature Review,” Journal of Biomedical Informatics 43, no. 3 (2010): 451–467. 20034594 10.1016/j.jbi.2009.12.004 PMC2878905

[53] Y. Ni , J. Wright , J. Perentesis , et al., “Increasing the Efficiency of Trial‐Patient Matching: Automated Clinical Trial Eligibility Pre‐Screening for Pediatric Oncology Patients,” BMC Medical Informatics and Decision Making 15, no. 1 (2015): 28. 25881112 10.1186/s12911-015-0149-3 PMC4407835

[54] S. S. Sahoo , S. Tao , A. Parchman , et al., “Trial Prospector: Matching Patients With Cancer Research Studies Using an Automated and Scalable Approach,” Cancer Informatics 13 (2014): 157–166. 25506198 10.4137/CIN.S19454 PMC4259509

[55] M. Wang , L. Dolovich , A. Holbrook , and S. M. Jack , “Factors That Influence Community Hospital Involvement in Clinical Trials: A Qualitative Descriptive Study,” Journal of Evaluation in Clinical Practice 28, no. 1 (2022): 79–85. 34008258 10.1111/jep.13583

