# Supplementary material for: “The Truth Is, We Must Miss Some”: A Qualitative Study of the Patient Eligibility Screening Process, and Automation Perspectives, for Cancer Clinical Trials
Source: Cancer Med. 2024 Dec 3;13(23):e70466. doi: 10.1002/cam4.70466 (PMC11612666; doi:10.1002/cam4.70466)
Supplement: Supplementary file 1 — Data S1. [file CAM4-13-e70466-s001.docx]

**Guide d’entretien**

Mené par Ambre La Rosa, enregistré par audio.

**Introduction**

Bonjour, Merci d’avoir accepté de participer à cet entretien.

Présentation intervieweur. Présentation du projet et de ses objectifs.

Si vous êtes d’accord, cet entretien va être enregistré et retranscrit. Vos données seront anonymisées.

1. **Présentation**

Pouvez-vous spécifier le nom de votre fonction ?

Depuis combien de temps exercez-vous cette fonction ?

En quoi consiste votre rôle par rapport à l’identification des patients éligibles au sein d’un essai clinique ?

À combien d’essais cliniques avez-vous participé depuis le début de votre fonction ?

1. **Processus d’identification des patients : exemple concret d’un essai clinique**

*Il est demandé au/à la participant.e de choisir un projet auquel il/elle a participé. Le projet doit être un essai clinique en oncologie. Le projet peut indifféremment avoir été terminé, abandonné ou en cours.*

- 1. **Présentation générale de l’essai** **clinique**

Pouvez-vous m’expliquer dans les grandes lignes l’essai en question ? (phase I-IV, but, essai uni ou multicentrique...)

Qui a initié l’essai ?

A t’il été abouti ?

- 1. **Processus d’identification des patients**

Combien de sujets nécessaires avaient été prévus ? Combien ont été inclus ?

Combien de temps a duré l’inclusion ? Le calendrier d’inclusion a-t-il respecté ? Si non, pour quelle raison ? Y’a-t-il eu un impact sur le respect du calendrier global de l’essai ?

Comment avez-vous identifié les patients éligibles à l’inclusion (quel personnel, par quels outils) ?

Comment avez-vous choisi le site de l’étude ?

Un logiciel d’aide à l’identification a-t-il été utilisé ? Si oui, en avez-vous vu le bénéfice ?

Autre modalité d’aide à l’identification ?

Une étude de faisabilité avait-elle été réalisée ?  Si non, en voyez-vous l’utilité a posteriori ?

- 1. **Détail sur logiciel d’aide à l'identification ou autre modalité d’identification**

Utilisez-vous un logiciel d’aide à l’identification des patients éligibles dans les essais ?

- Si oui, quel est-il ?

Depuis quand l’utilisez-vous ? Savez-vous par qui il a été développé ou comment il a été choisi ?

Concrètement, quel type de personnel l’utilise ? (médecins ? ARCs ? URC ?) Pour quel type d’étude ?

Comment fonctionne-t-il ? Si vous faites partie de l’utilisateur cible, pouvez-vous me montrer un exemple ?

L’interfaçage se fait-il directement depuis le dossier patient ou une autre source de données (registre, PMSI...) ? Quelles données traite-t-il ?

Quels sont les points de satisfaction et/ou d’insatisfaction vis-à-vis de ce logiciel ?

*Pistes de réflexion : performance, fonctionnalités, latence éventuelle, interface)*

Est-il difficile à utiliser ? Avez-vous eu besoin ou recommanderiez-vous une formation ?

Est-il inutilisable dans certains cas d’essais cliniques ou de données ?

Quel a été l’impact du déploiement du logiciel sur l'identification des patients éligibles dans vos essais dans vos essais cliniques ?

- Sinon, comment procédez-vous ?

Quelles données utilisez-vous pour évaluer l’éligibilité des patients ? (Registre, DPI, PMSI...)

Est-ce possible d'envoyer des données à d'autres institutions et d'en recevoir d'elles ? (par quel outil)

Y-a-t-il des contraintes réglementaires spécifiques ?

Quant aux études de faisabilité, en faites-vous pour chaque essai clinique ? Sinon, à quelle fréquence ?

Comment et sur quel type de données les réalisez-vous ? (manuellement depuis DPI, EDS, automatiquement..)

1. **Généralisation**

De manière générale, l’identification des patients éligibles se passe-t-elle toujours de cette façon ? Sinon, autre modalité ?

Y-a-t-il des modalités spécifiques selon la phase ? Ou un type de pathologie ?

Qu'en est-il du choix des centres d’inclusion ?

Comment se passe les études multicentriques ? Connaissez-vous d’autres modalités dans d’autres centres ? Quelle interopérabilité ?

Avez-vous noté une amélioration quant à l’identification des patients éligibles au cours de ces dernières années ? Qui a été moteur ? Quels obstacles avez-vous rencontré ?

A l’état actuel, quelles problématiques identifiez-vous ?

Voyez-vous des pistes d’amélioration ? Si pas de logiciel, en voyez-vous l’utilité ?

1. **Conclusion**

Merci pour votre temps.

Souhaitez-vous être averti de la publication des résultats de cette étude ?
